# Nervous Necrosis Virus (NNV) Booster Vaccination Increases Senegalese Sole Survival and Enhances Immunoprotection

**DOI:** 10.3390/ani13010051

**Published:** 2022-12-23

**Authors:** Carmen López-Vázquez, Sandra Souto, José G. Olveira, Ana Riaza, Óscar González, Cristina Brea, Alejandro M. Labella, Dolores Castro, Isabel Bandín

**Affiliations:** 1 Departamento de Microbiología y Parasitología, Instituto de Acuicultura, Universidade de Santiago de Compostela, 15782 Santiago de Compostela, Spain; 2 Stolt Sea Farm, Edificio Quercus, 15707 Santiago de Compostela, Spain; 3 Departamento de Microbiología, Instituto de Biotecnología y Desarrollo Azul (IBYDA), Universidad de Málaga, 29071 Málaga, Spain

**Keywords:** Senegalese sole, nervous necrosis virus (NNV), re-immunization, humoral response, immune-related genes expression

## Abstract

**Simple Summary:**

Viral encephalopathy and retinopathy (VER), caused by nervous necrosis virus (NNV), is a serious threat to Senegalese sole farming. We have previously demonstrated that immunization with an inactivated vaccine confers partial protection against the infection. However, a vaccination program must be finely adjusted to achieve the best results in terms of immune system stimulation and protection. In this study we show that a booster injection 30 days after prime vaccination increases sole survival and reduces NNV replication in brain (viral target organ). The analysis of immune-related genes expression indicated that T CD4+ lymphocytes and the proteins Mx and HERC4 may play an important role in the protection. These findings increase our understanding of sole immune response against NNV and may contribute to the development of effective protection measures.

**Abstract:**

A re-immunization programme has been tested to improve the protective response elicited in sole by a previously developed BEI-inactivated betanodavirus vaccine. The vaccine was prepared using a reassortant RGNNV/SJNNV strain which is highly pathogenic for sole, and vaccination assays were performed by intraperitoneal injection. Experimental design included a prime- and a booster-vaccination group, which consisted of individuals that received a second vaccine injection at 30 days post vaccination), and their respective controls. A month after prime/booster vaccination, fish were challenged by intramuscular injection with the homologous NNV strain. Samples were collected at different times post vaccination and post challenge to assess the immune response and viral replication. Booster dose enhanced the protection against NNV infection because a significant increase in survival was recorded when compared with prime-vaccinated individuals (relative percent survival 77 vs. 55). In addition, a clear decrease in viral replication in the brain of challenged sole was observed. During the immune induction period, no differences in IgM production were observed between prime- and booster-vaccinated fish, and the expression of the antigen presenting cells (APC)-related molecule MHC class II antigen was the only differential stimulation recorded in the re-immunized individuals. However, a significant upregulation of *mhcII* and the lymphocytes T helper (Th) marker cd4 was observed after the challenge in the booster-vaccinated group, suggesting these cells play a role in the protection conferred by the booster injection. In addition, after viral infection, re-immunized fish showed specific and neutralizing antibody production and overexpression of other immune-related genes putatively involved in the control of NNV replication.

## 1. Introduction

Nervous necrosis virus (NNV), the causative agent of viral encephalopathy and retinopathy (VER), is one of the most widespread fish viruses, being detected in more than 170 species [1]. NNV is a small non-enveloped virus member of the genus *Betanodavirus* within the family *Nodaviridae*. Its genome is composed of two single stranded RNA segments named RNA1, which codes for the RNA-dependent polymerase (RdRp), and RNA2, encoding the capsid protein (CP). Based on a small variable sequence of RNA2, namely the T4 region, betanodaviruses are classified into four genotypes: barfin flounder, red spotted grouper-, stripped jack-, and tiger puffer nervous necrosis virus (BFNNV, RGNNV, SJNNV, and TPNNV, respectively). For years, the RGNNV genotype was the main concern for Mediterranean aquaculture because of the high susceptibility of European sea bass, one of the most economically important farmed species in the region. However, the emergence in Southern Europe of natural reassortants between the RGNNV and SJNNV genotypes has been reported as a threat for Senegalese sole and gilthead seabream farming [2,3,4,5].

NNV-infected fish show abnormal swimming behaviour and anorexia as the main clinical signs resulting from lesions caused to the central nervous system [1]. The most affected growth stages are larvae and young juveniles with mortality rates ranging between 80–100%, but outbreaks have also been reported in larger fish [6,7,8,9]. Viral transmission occurs both horizontally (through the water column, fish-to-fish or through invertebrate vectors) and vertically (the virus has been detected in the sexual products of both male and female adult fish) [10,11].

Different strategies have been implemented to avoid NNV infection in larval fish, including the selection of NNV-free broodstocks and disinfection of fertilised eggs [10]. However, an effective vaccination program to prevent viral outbreaks is desirable, especially if it can be applied to fish of different sizes. Several NNV experimental vaccines have been developed for different fish species including inactivated vaccines for grouper, Asian and European sea bass [12,13,14,15,16,17,18], subunit vaccines for turbot and Atlantic halibut, groupers and European sea bass [19,20,21,22,23,24,25], and DNA vaccines for grouper and Asian sea bass [26,27].

Our group has previously reported that a non-adjuvanted binary ethylenimine (BEI)-inactivated vaccine prepared with a reassortant NNV strain and administered by intraperitoneal injection in juvenile Senegalese sole stimulated an immune response and partially improved survival (RPS 51%) [28]. Insufficient protection is the most common problem in vaccine development and can be attributed to the failure of the induction of both humoral and cellular immunity [29,30]. Boosting has been regarded as a useful tool to enhance viral vaccine performance [31,32,33]. Our preliminary results indicated that time between prime vaccination and booster was a determining factor in the improvement of vaccine performance [34]. Therefore, in order to improve the protective response elicited in sole by the BEI-inactivated vaccine, a vaccination program including a booster injection at 30 days post prime vaccination has been tested. The level of protection in the re-immunised group was compared with that of the prime-vaccinated group. In addition, antibody response and expression profiles of immune-related genes of vaccinated and challenged fish was assessed.

## 2. Materials and Methods

### 2.1. Viral Strain and Propagation

NNV strain SpSsIAusc160.03 (hereafter Ss160.03), a reassortant RGNNV/SJNNV (according to the RNA1 and RNA2 of the parental strains) obtained from a VER episode in farmed Senegalese sole [2], was used for both vaccine preparation and the viral challenge. The strain was grown in E-11 cells, clone-derived from the SSN-1 cell line [35], maintained in L-15 Leibovitz medium (Lonza) supplemented with penicillin (100 units/mL), streptomycin (100 mg/mL), and 2% foetal bovine serum (FBS, Lonza) at 25 °C. E-11 cells were also used for viral titration and neutralization assays.

### 2.2. Vaccine Preparation

The BEI inactivated vaccine was prepared as previously described [28]. Briefly, a suspension of the Ss160.03 strain with a titre of 3 × 10^8^ TCID_50_/mL was inactivated by mixing with freshly prepared BEI solution at 1 mM final concentration, for 72 h at 25 °C. The inactivation was confirmed by the absence of CPE and viral titre in the E-11 cell line, after three 10-day blind passages.

### 2.3. Fish Vaccination

**Figure 1:**
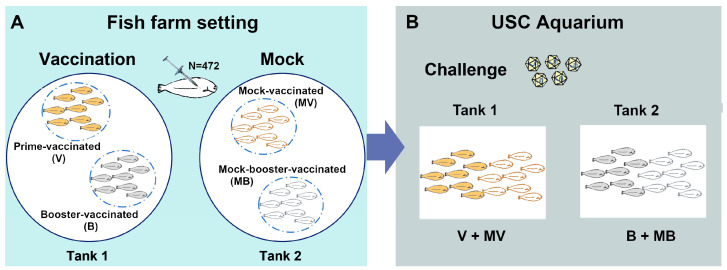
Vaccination and challenge conditions. Fish vaccination was performed in a fish farm (**A**). After prime vaccination or booster, fish were transported to the USC aquarium facilities for challenge (**B**).

Vaccination assays were performed at the experimental facilities of a fish farm (Stolt Sea Farm, Ribeira, Spain). NNV free-Senegalese sole juveniles (average weight 3 g) were maintained at 20 °C in opaque tanks containing sea water (35 ppt-salinity and oxygen content higher than 8 mg/L) and fed with commercial diets routinely used in the farm. At the farm facilities, fish (N = 472) were equally distributed in two tanks, one used for the vaccination assay and the other as a control (N = 236/tank). Sole individuals were anaesthetized with 75–100 mg/L of tricaine methanesulfonate (MS-222, Sigma-Aldrich, St. Louis, MO, USA) and intraperitoneally (ip) injected with 100 µL of the BEI-inactivated vaccine (10^7^ TCID_50_/mL) or PBS. After the initial injection, half of the vaccinated/control fish received a second injection (booster injection) at 30 days post vaccination, resulting in 4 experimental groups (N = 118, Figure 1A): prime-vaccinated (V) and booster-vaccinated fish (B), and the controls mock-vaccinated (MV) and mock-booster-vaccinated fish (MB). Fish that received a second dose (B and MB) were marked in the abdominal area with visible implant elastomer (VIE) tags (Northwest Marine Technology, Inc., Anacortes, WA, USA).

**Figure 2:**
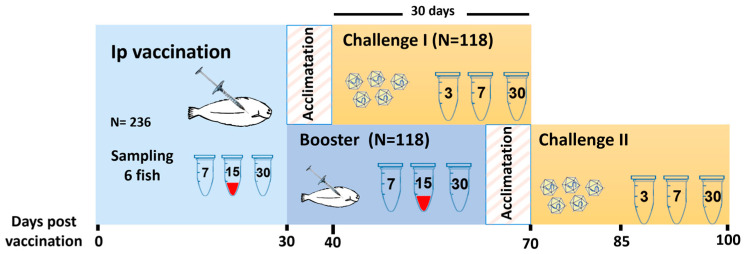
Experimental design. After arrival to the USC aquarium facilities, fish were acclimated for 10 days before challenge. Mock-vaccinated individuals were handled and sampled as vaccinated fish.

Fish (n = 6 per group and time point) were sampled at 7- and 30-days post prime- or booster-vaccination (dpv and dpb, respectively) for blood and head-kidney collection, and at 15 dpv/dpb only for blood extraction (Figure 2).

### 2.4. NNV Challenge

At 30 days post prime- or booster-vaccination, fish were transferred from the farm to the aquarium facilities of the University of Santiago de Compostela. Average weight of sole individuals was 6 g for prime-vaccinated fish, and 10 g for those animals re-immunized at 30 dpv. Vaccinated and control sole from each experimental group (prime- and booster-vaccinated) were placed in the same 300-L tanks containing sea water and acclimated to increasing temperature for 10 days until reaching 22 °C (Figure 1B). Then, at 40 dpv/dpb, fish were anaesthetized as previously described and experimentally infected by intramuscular injection of the homologous viral strain Ss160.03, at a dose of 2 × 10^4^ TCID_50_/g. At 3-, 7-, and 30-days post challenge (dpc), six fish from each group were sampled for immunological analysis (Figure 2). Additionally, to monitor viral replication after challenge in the different experimental groups, NNV load in the brain of the infected fish was assessed at different time points (3, 7, 15, 23, and 30 dpc).

### 2.5. Fish Sampling

For serum obtention, fish were bled in the caudal peduncle and blood was incubated overnight at 4 °C. After centrifugation at 10,000× *g* for 10 min at 4 °C, serum samples were collected and immediately stored at −20 °C. For tissue sampling, fish were sacrificed with an MS-222 overdose, and head-kidney and brain were aseptically removed and immediately stored at −80 °C.

### 2.6. Specific and Neutralizing Antibody Levels

Antibody detection was performed as previously described [28]. Specific NNV-antibodies (IgM-NNV) were detected using an indirect ELISA. All assays were performed in duplicate and positive (previously assayed positive serum) and negative controls were included. Briefly, total proteins from serum samples obtained from vaccinated (7, 15, and 30 dpv/dpb) and challenged fish (3, 7, and 30 dpc) were incubated overnight at 4 °C, washed with PBST (PBS with 0.2% of Tween-20) and blocked with 5% skimmed milk in PBST. Afterwards, 0.1 mL of NNV suspension (Ss160.03 strain, 2.5 × 10^6^ TCID_50_/mL) was added into precoated and blocked wells. After incubation at room temperature, plates were washed with PBST and incubated for 2 h at room temperature firstly with a rabbit anti-NNV serum (Abcam, Inc.; 1:10,000), and then with the anti-rabbit IgG-HRP (Sigma Aldrich; 1:25,000, St. Louis, MO, USA). The reaction was revealed with 3,3′,5,5′-tetramethylbenzidine single solution (ThermoFisher, Vilnius, Lithuania) and the absorbance was read at 450 nm with an iMark™ Microplate Absorbance Reader (BioRad, Hercules, USA).

For the detection of neutralizing antibodies, serial dilutions of sera (decomplemented at 56 °C for 30 min) obtained from vaccinated and challenged fish at the time points indicated above were incubated with equal volumes of 10^2.5^ TCID_50_/mL of Ss160.03. After 1 h incubation at 25 °C, samples were assayed for NNV replication on E-11 cells and the serum dilutions which provoked the absence of CPE were determined. Serum from an infected fish served as a positive control.

### 2.7. Gene Expression by Real-Time Polymerase Chain Reaction

The procedures of RNA extraction, reverse transcription, and real-time PCR are described in a previous vaccination study [28]. Briefly, total RNA was isolated from head-kidney samples using a Nucleospin RNA II kit (Macherey-Nagel, Düren, Germany), reverse transcription was carried out using Superscript IV (Invitrogen™, Vilnius, Lithuania) with Random Hexamers (ThermoFisher, Vilnius, Lithuania), and the expression of sole immune-related genes was analysed by RT-qPCR in an iCycler iQ CFX96™ Real Time System (BioRad). Reaction mixtures consisted of 2 μL of the cDNA template and 0.2 μM of the specific primers in 20 μL final volume using iQ™ SYBR Green supermix (BioRad Tower, Singapore). Amplification protocol included an activation/denaturation step for 3 min at 95 °C, followed by 40 cycles of 15 s at 95 °C and 30 s at 55 or 58 °C. Negative controls were always included in the reactions. The specific primers used are shown in Table 1. Relative gene expression was calculated by the 2^−ΔΔCt^ method [36] and the β actin (*actb*) gene was used as the endogenous control.

### 2.8. Viral Quantification

NNV RNA1 extraction and amplification was performed as described above using NodR1 primers following the validated method described by [39]. All samples were tested in triplicate. Quantification was accomplished using a standard curve of a plasmid DNA containing the full-length RNA1 of strain Ss160.03 (20-fold dilutions from 10^1^ to 10^7^ copies/μL). Viral load data were expressed as RNA1 copies per g of fish tissue.

### 2.9. Calculations and Statistics

The relative percent survival (RPS) was calculated from the cumulative mortality using the following formula:

$$\mathrm{RPS}=\left[1-\frac{\%\;\mathrm{mortality}\;\mathrm{of}\;\mathrm{vaccinated}\;\mathrm{group}}{\%\;\mathrm{mortality}\;\mathrm{control}\;\mathrm{group}}\right]\times \;100$$

## 3. Results

### 3.1. Antibody Production after Prime- and Booster-Vaccination

**Figure 3:**
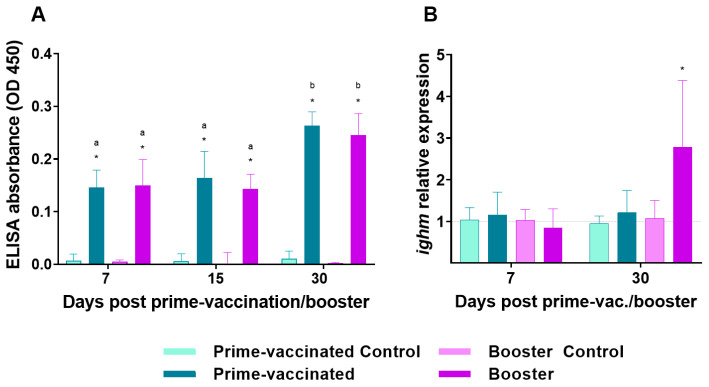
IgM levels in the serum (**A**) and transcription of *ighm* (**B**) in the head-kidney of prime-vaccinated and re-immunized fish at 7-, 15- and 30-days post vaccination/re-immunization. Data represent the mean ± standard deviation (n = 6 fish/group and time). Asterisks represent statistical differences between control and prime-vaccinated/booster groups (*p* < 0.05). Significant differences between groups are indicated by different lower letters over bars (*p* < 0.05).

Significant production of specific antibodies was detected by the ELISA assay after 7 days in prime- and booster-vaccinated fish and the IgM response increased over time in a similar way in both groups (Figure 3A). However, no neutralizing antibodies were detected. 

To further analyse the antibody response, transcriptional levels of *ighm* gene in the head-kidney were assessed at 7 and 30 dpv/dpb. After 30 dpv/dpb a significant increase in the *ighm* transcriptional levels was recorded in fish from the booster group, which differed significantly from prime-vaccinated individuals (Figure 3B). 

### 3.2. Expression of Immune Genes in Vaccinated Fish

**Figure 4:**
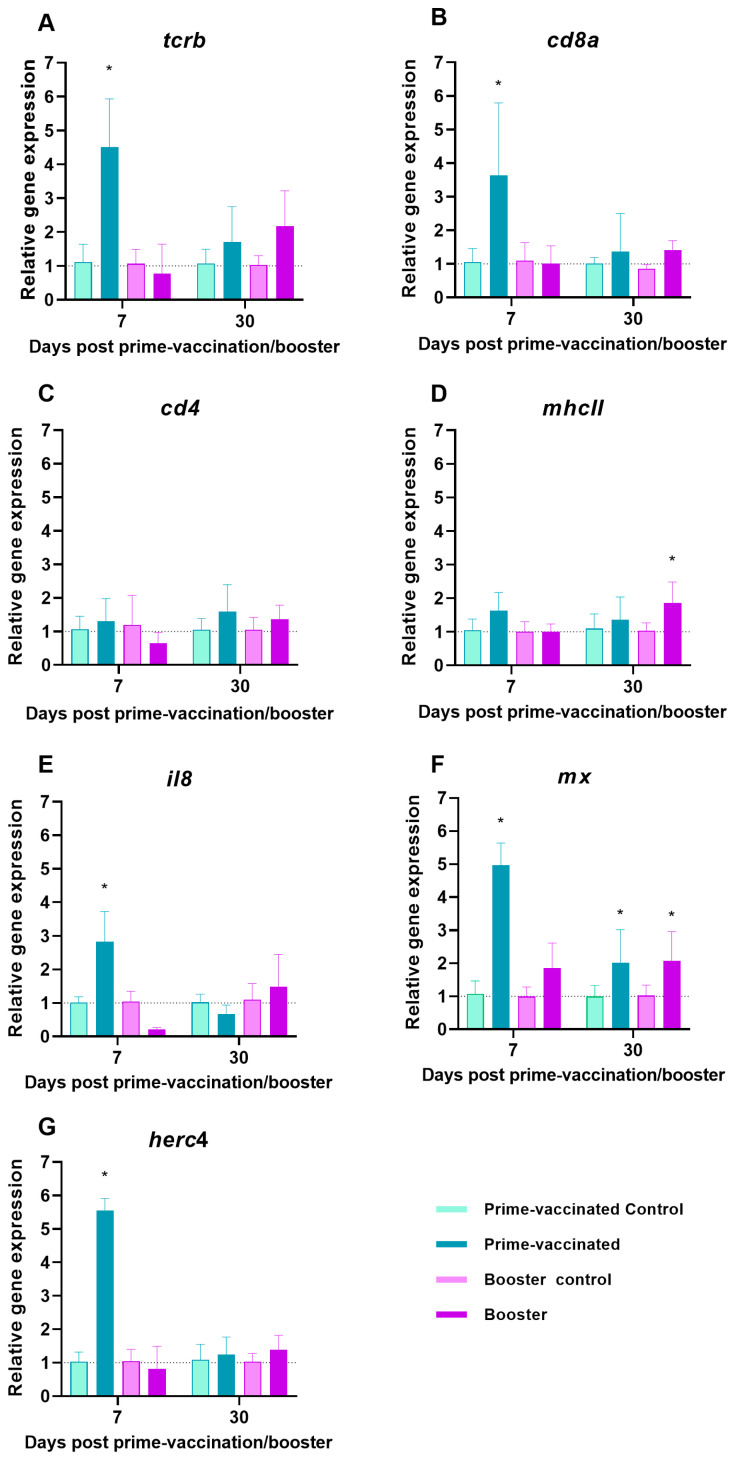
Expression of immune-related genes (**A**) *tcrb*, (**B**) *cd8a*, (**C**) *cd4,* (**D**) *mhcII*, (**E**) *il8*; (**F**) *mx* and (**G**) *herc4*, in the head-kidney of Senegalese sole specimens 7- and 30-days after intraperitoneal vaccination or re-immunization with a BEI-inactivated vaccine or PBS (control). Data represent the mean ± standard deviation (n = 6 fish/group and time). Asterisks represent statistical differences between control and prime-vaccinated/booster groups (*p* < 0.05).

At 7 dpv a significant up-regulation in the mRNA expression of cytotoxic T lymphocytes (CTL)-related molecules TCRβ and CD8α (*p* < 0.0001) was observed in prime-vaccinated fish (Figure 4A,B). In addition, overexpression of *il8*, *mx* and *herc4* genes was also detected (Figure 4E–G). However, a decay to control levels was noticed at 30 dpv in all cases, excepting for *mx* (Figure 4A,B,E–G). No modulation of the levels of T helper (Th)-related molecule CD4 and the antigen presenting cells (APC)-marker MHCII was observed (Figure 4C,D). In the booster-vaccinated fish only moderate but significant overexpression of *mhcII* and *mx* (*p* < 0.02) was recorded at 30 dpb (Figure 4D,F). 

### 3.3. Efficacy Protection of Prime- and Booster-Vaccination

**Figure 5:**
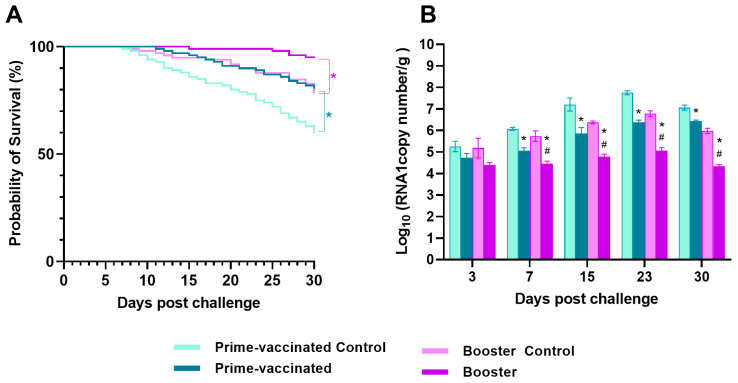
Protection conferred by the vaccination/re-immunization and decrease of the viral load in sole brains. (**A**) percent of survival during 30 days of in vivo infection and (**B**) viral load (n =3 fish/group and time) in brain from challenged *Senegalese sole* specimens. (**A**) Survival rates were compared between groups using the Kaplan Meier test. Asterisks denote differences between survival curves according to a log-rank Mantel Cox test (*p* < 0.001), (**B**) Infection was performed by intramuscular injection with 2 × 10^4^ TCID_50_/g. Asterisks represent statistical differences between control and prime-vaccinated/booster groups whereas hash symbols represent significant differences between prime- and booster-vaccinated groups at a same time point (*p* < 0.05).

A month after vaccination/booster, fish were challenged by intramuscular injection with the homologous NNV strain. Control fish from the prime-vaccination experiment started to show typical VER signs (mainly changes in swimming behaviour and anorexia) at 5 dpc and suffered the first mortalities at 7 dpc, whereas the onset of clinical signs and mortalities in the vaccinated fish were delayed until 9 and 11 dpc, respectively. As for the booster-vaccination assay fish, the mock-immunized fish started to show disease signs at 7 dpc and mortality one day later, whereas in the re-immunized fish, disease signs and mortality were delayed to 12 and 15 dpc, respectively. At 30 dpc, a significant improvement of survival was observed in both groups (*p* < 0.005) (Figure 5). although differences were appreciated when compared with the survival in the respective mock vaccinated groups. Thus, whereas in prime-vaccinated fish the relative percent of survival (RPS) was 55% in the booster-vaccinated group, RPS increased to 76% (Table 2, Figure 5A).

### 3.4. Progression of the Viral Load in the Vaccinated and Re-Immunized Fish after Challenge

To monitor viral replication after challenge in the different experimental groups, NNV load in the brain of the infected fish was assessed at different time points (3, 7, 15, 23, and 30 dpc). At 3 dpc the viral load was very similar in the prime- and booster-vaccinated fish, and no significant differences were found between immunized and mock-immunized fish. At 7 dpc an increase in the viral load was observed in all experimental groups with moderate but statistically significant differences between prime- and booster-vaccinated fish and their respective control groups (0.5 and 0.78 log, respectively) (Figure 5B). As viral load increased, differences between immunized and mock-vaccinated fish in both experimental groups also increased (around 1.5 logs in both assays). However, at day 30 the difference in the prime-vaccination experiment was only 0.63 log, whereas in the booster assay it was higher than 1.5 log.

### 3.5. Antibody Response in Challenged Fish

**Figure 6:**
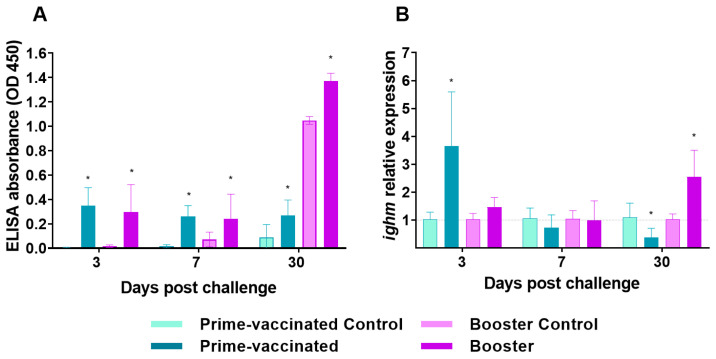
IgM levels in the serum (**A**) and transcription of *igm* (**B**) in the head-kidney of vaccinated and re-immunized fish at 3-, 7-, and 30-days post challenge (dpc). Challenge was performed by intramuscular injection with 2 × 10^3^ TCID_50_/g for prime- and booster-vaccinated fish, respectively. Data represent the mean ± standard deviation (n = 6 fish/group and time). Asterisks represent statistical differences between control and prime-vaccinated/booster groups (*p* < 0.05).

The in vivo infection elicited a significatively higher antibody titres in vaccinated fish compared to control fish, being the higher titres recorded at 30 dpc in booster-vaccinated fish (Figure 6A). In addition, neutralizing antibodies were detected at 30 dpc only in the booster-vaccinated individuals, with a titre of 216.

A different modulation of *ighm* gene expression was recorded in the head-kidney of the immunized groups after challenge (Figure 6B). At 3 dpc a significant up-regulation was observed in prime-vaccinated fish followed by a decay to values similar to control levels, whereas in the booster-vaccinated fish the increase in the *ighm* transcriptional levels was observed only at 30 dpc.

### 3.6. Immune Gene Expression after Challenge

**Figure 7:**
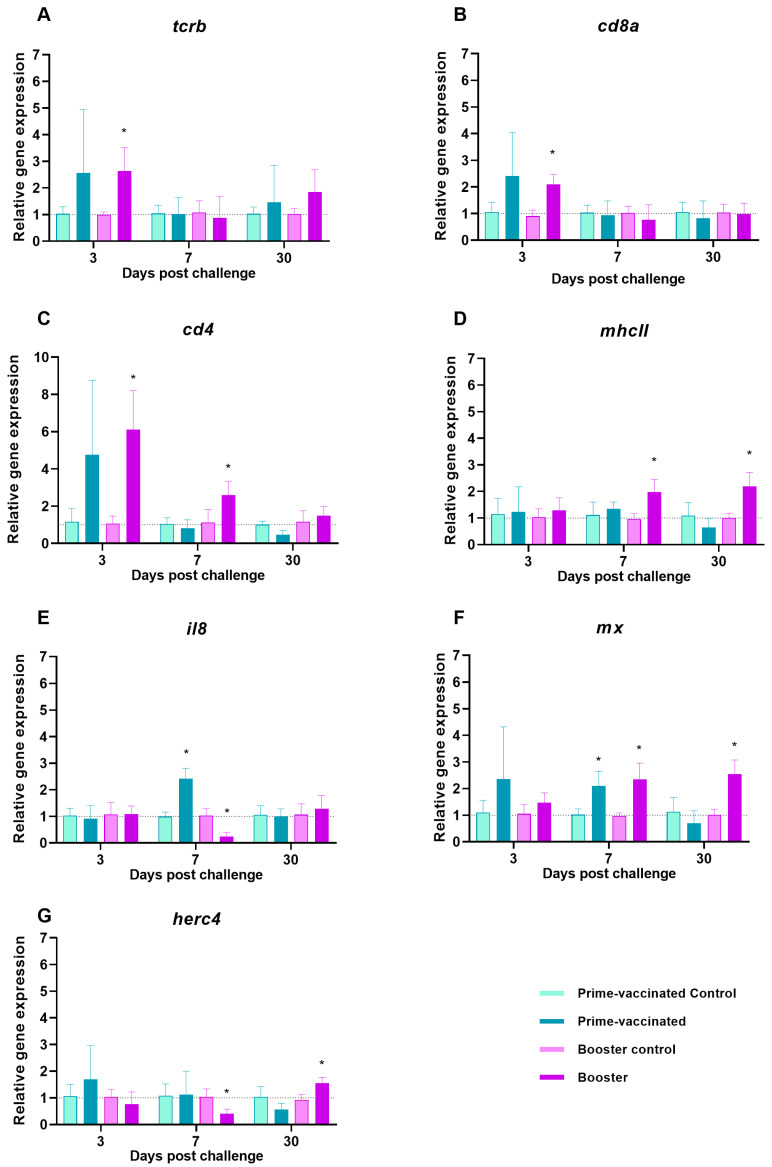
Expression of immune-related genes (**A**) *tcrb*, (**B**) *cd8a*, (**C**) *cd4,* (**D**) *mhcII*, (**E**) *il8*; (**F**) *mx* and (**G**) *herc4*, in the head-kidney of Senegalese sole specimens at 3-, 7-, and 30-days post challenge (dpc). Challenge was performed by intramuscular injection with 2 × 10^3^ TCID_50_/g for prime- and booster-vaccinated fish, respectively. Data represent the mean ± standard deviation (n = 6 fish/group and time). Asterisks represent statistical differences between control and prime-vaccinated/booster groups (*p* < 0.05).

In prime-vaccinated fish, significant up-regulation was only recorded in *il8* and *mx* genes at 7 dpc (Figure 7E,F). However, in the booster-vaccinated individuals a significant overexpression of most genes was observed at different time points after challenge. An up-regulation of CTL-related genes was recorded at 3 dpc (Figure 7A,B), decaying to control levels afterwards. Besides, overexpression of the Th cell marker *cd4* was observed at the beginning of the challenge (3 and 7 dpc) (Figure 7C), whereas induction of *mhcII* and *mx* expression were observed later (7 and 30 dpc) (Figure 7D,F). No induction of *il8* was detected at any time, on the contrary, down-regulation was observed at 7 dpc (Figure 7E). It should be noted that the increase in the transcripts of *herc4* at 30 dpc was preceded by a down-regulation at 7 dpc (Figure 7G).

## 4. Discussion

The main goal of vaccination is to induce a protective response in the host species against a pathogen [40]. We have previously reported that a BEI-inactivated vaccine induced an immune response but only partial protection (RPS = 51) against NNV infection in juvenile sole when administered by intraperitoneal injection [28]. In the present study, a re-immunization program including a booster injection at 30 dpv was assessed and our findings demonstrate that re-immunization significantly enhances protection. At the end of NNV challenge (30 dpc), booster-vaccinated fish showed a high survival (~95%) which differed significantly from that of the prime-vaccinated sole (*p* = 0.0021). As it has been widely reported in the literature that the youngest fish are the most susceptible to NNV infection, we conducted the experiments with juveniles that reached the minimum size to be safely injected. Susceptibility at these developmental stages has been demonstrated by the high mortality rates achieved in the control groups.

The relative percent survival was also clearly higher in the re-immunized individuals than in the vaccinated ones (76 vs. 55). Moreover, the booster provoked a significant decrease in the viral load detected by RT-qPCR in the brain of challenged fish when compared with the mock-boostered animals from 7dpc onwards (higher than 1.5 log from day 15). Although a reduction in the viral load of prime-vaccinated fish was also observed from 7 dpc, it was lower than that observed in the booster assay, and at 30 dpc the difference with the mock-vaccinated fish was reduced to only 0.6 log.

The differences in survival and viral load between prime- and booster-vaccinated fish cannot be correlated with an increase in the humoral response during the immune induction period, because although specific IgM-NNV production was elicited after the booster injection, the overall antibody levels were similar in the prime-vaccinated and re-immunized groups.

Although a significant increase in the transcriptional levels of the *ighm* gene was observed in the head-kidney of booster-vaccinated fish at 30 dpb, suggesting that a further increase in the circulating IgM levels could come about, the IgM production recorded in challenged fish does not support this hypothesis. A possible explanation for this lack of correlation could be the detection of sterile transcripts that do not produce the corresponding peptide. The role of the anterior kidney in humoral memory has already been highlighted [41,42] and, although the brain is the target tissue for NNV infection, in the present study this lymphoid organ was chosen for immune response assessment because no relevant immune-related gene stimulation was recorded in the brain of BEI-vaccinated sole [28]. No neutralizing antibodies were detected in either prime- or booster-vaccinated fish, which contrasts with the results obtained in NNV-booster vaccinated Asian seabass (*Lates calcarifer*) in which a high antibody neutralizing activity has been related to the vaccination protection efficacy [43].

Ruling out the involvement of humoral immunity in the enhancement of protection caused by the booster injection, the activation of cellular immunity must be considered. Cytotoxic T lymphocytes (CTLs) have been regarded as the primary players in antiviral adaptative immunity in teleost fish (reviewed by [44]). Although both *cd8* and *tcrb* genes were upregulated at 7 dpv in prime-vaccinated fish, the stimulation was not maintained at 30 dpv. However, the booster-vaccinated fish did not show modulation of either of those genes regardless of the time point analysed. A similar induction pattern to that observed in the prime-vaccinated fish was reported in previous NNV vaccination studies performed with giant grouper (*E. lanceolatus*), which showed *cd8* gene expression differentially regulated depending on the vaccine concentration at 7 and 15 dpv but not significantly modulated at 30 dpv [45]. As no immunogen response has been analysed in booster vaccinations against NNV to date, we can only compare our results with a booster vaccination assay performed against viral haemorrhagic septicaemia virus (VHSV) in Olive flounder [46]. In that study a significant increase in the transcriptional levels of the *mhcII* gene, which was correlated with the activation of CD8+ T cells, was recorded after 48 h, but the values returned to control levels at 30 dpb. Although no modulation of the transcriptional level of lymphocyte markers was observed in the booster-vaccinated sole, we did observe an upregulation of *mhcII* gene expression at 30 dpb. Antigen presentation is an essential step in the induction of virus-specific immunity by the adaptive immune system [47]. Viral particles phagocytosed and/or endocytosed by antigen presenting cells (APC) are presented in association with MHC class II molecules to naïve CD4+ T cells [48]. In the already cited booster vaccination assay against VHSV, a significant increase in the transcriptional levels of *mhcII* was also observed, which was correlated with an increase in CD4+ cells [46]. However, in a previous study on cellular immune response in rainbow trout following vaccination and challenge against salmonid alfavirus (SAV), no correlation between *mhcII* and *cd4* gene expression was recorded [49]. According to these authors, this could be explained by different APC types involved in the immune response with different *mhcII* regulation.

An inflammatory reaction, critical to the efficiency of the immune response to viral infections, is characterized by the systemic release of specific cytokines and chemokines such as IL8/CXCL8 and by the chemotactic migration of leukocytes to the site of inflammation [50]. In our study, whereas in prime-vaccinated fish a significant upregulation of *il8* was recorded at 7 dpv, no modulation was observed in booster-vaccinated fish. An increase in *il8* expression has been reported in salmonids infected with VHSV, salmonid alfavirus 3 (SAV3), and IPNV [50,51,52], and in turbot (*Scophthalmus maximus*) inoculated with poly (I:C) [53]. Although the role of IL8 in the adaptive immune response to fish infections is not clear [54], the *il8* gene has been reported to be upregulated by bacterial vaccination [53,55].

Contrary to what our group has previously reported [28], the *herc4* gene was upregulated in the head-kidney of prime-vaccinated fish at 7 dpv, but no gene expression modulation was observed in the booster-vaccinated group. However, at 7 dpc, *herc4* downregulation was recorded only in booster-vaccinated fish. HERC4 is a ligase of the G protein-coupled receptor Smoothened (Smo), a key signal transducer of the Hedgehog (HH) signalling pathway [56]. Recent evidence has shown that HH signalling is targeted by several human viruses to facilitate viral transcription and immune evasion leading to effective viral replication and pathogenesis [57]. The *herc4* gene is upregulated in NNV-infected fish, whereas it is inhibited in asymptomatic carriers [38,58], suggesting that it may be involved in the control of the viral infection. At 30 dpc, a moderate but significant upregulation was observed. Since all previous studies on this gene were performed at early times post-infection/stimulation, no long-term information is available. In addition, as our experiment ended at 30 dpc, we cannot predict if this overexpression would influence fish immune response. The antiviral Mx protein coding gene was also upregulated after infection in prime- and booster-vaccinated fish at 7 dpc, but the induction was only maintained at 30 dpc in booster-vaccinated individuals in which the viral load showed the highest difference with the control group, supporting that Mx is involved in the control of NNV replication, as previously reported in grouper [59].

IgM production was observed early (3 dpc) in both prime- and booster-vaccinated fish after NNV challenge, but at 30 dpc *ighm* transcriptional levels in the head-kidney, as well as antibody levels, were significantly higher in the re-immunized individuals. These findings indicate that the booster injection triggered a more sustained humoral response over time than prime-vaccination, which is reinforced by the fact that neutralizing antibodies were first detected in booster-vaccinated fish at 30 dpc. Although CTL modulation was not observed after booster injection, *cd8a* and *tcrb* transcriptional levels were slightly but significantly upregulated after challenge (3 dpc) in the booster-vaccinated fish, going down to control levels afterwards. A similar CD8α induction pattern has been observed in vaccinated groupers after NNV challenge [45] and cell-mediated cytotoxicity against carp hematopoietic necrosis virus (CHNV) has been recorded up to 7 days in crucian carp (*Carassius auratus langsdorfii*) after booster immunization [60]. The upregulation of *mhcII* transcription recorded at the end of the immune induction period in booster-vaccinated fish was also observed after NNV challenge (from 7 dpc until the end of the experiment). These findings agree with the *mhcII* induction observed in bath and orally NNV-vaccinated grouper larvae [17]. Furthermore, a significant increase in *cd4* transcriptional levels was observed up to 7 dpc in the re-immunized group, suggesting a putative role of Th cells in the adaptative response of sole to NNV infection. As observed in the induction period, *il8* upregulation was recorded in prime-vaccinated individuals at 7 dpc. On the contrary, a downregulation was recorded in booster-vaccinated fish. It has been suggested that the downregulation of pro-inflammatory cytokines at late time points after viral infection may be related to the onset of adaptive immunity [61]. In our study, *il8* gene expression was downregulated a week after the challenge. It could be speculated that this downregulation is related to the higher protection achieved in the booster assay by avoiding a post infection inflammatory response, which seems to play an important role in the pathology of NNV infection [62,63]. Further analysis of pro-inflammatory cytokines in prime-vaccinated and boostered individuals are necessary to confirm this hypothesis.

## 5. Conclusions

We have demonstrated that a booster injection 30 days after prime vaccination improves the performance of a BEI-inactivated vaccine against NNV infection in Senegalese sole. A significant increase in survival (RPS 77 vs. 55) and a clear decrease in viral replication in brain was observed when compared with prime vaccination. During the immune induction period, no differences in IgM production were observed between prime- and booster-vaccinated fish, and the expression of *mhcII* was the only differential stimulation recorded in the booster-vaccinated individuals. However, a significant induction of the mRNA expression of *cd4*, *mhcII,* and *mx* genes was observed after challenge in booster-vaccinated fish, which also showed production of neutralizing antibodies.

## Figures and Tables

**Table 1:** Primer sequence used for gene expression analysis.

Gene	Sequence 5′-3′	Accession No., Reference or Unigene ID
*cd4*	F: GACCTCAGGCTGCAATGGT R: TGAGCAGAGTGATGGACAGACT	[37]
*cd8a*	F: GTCGCAGTTCTGCTCTCCGC R: TCGGTTGCAGTAGAGGACGG	solea_v4.1_unigene59609 ^a^
*herc4*	F: GCCAAAACACTGGCATGGTT R: AACGCCAAACAGGAAGTACCT	[38]
*ighm*	F: TGAAACATTGACACAGCCAGCC R: CGTGTGAGCTTCCAATCCACTC	solea_v4.1_unigene691100
*il8*	F: AAGGTCCTTACTGCGCAAAC R: TGCTCTTCCCTGCTGATGAA	solea_v4.1_unigene18346
*mchII*	F: CGCTGATGAAAATGATCCACCTTCT R: ACCAGTCACATGACAGATCAGAGT	[38]
*mx*	F: CCTCTCTCCTTCAGGATCCTCCTCCTGTGC R: CAAAACAAGAAACTATCTGCCTGGTGGTTC	[38]
*tcrb*	F: CAGGAGGCACAGCTATGAAA R: TCTCCACCCAAATCTCCAAA	solea_v4.1_unigene681812
*actb*	F: GACGACATGGAGAAGATC R: GGTGTTGAAGGTCTCAAA	DQ485686 ^b^

^a^, https://www.scbi.uma.es/soleadb. ^b^, https://www.ncbi.nlm.nih.gov/nuccore/DQ485686 (accessed on 21 November 2022).

**Table 2:** Immunization conditions and conferred protection against NNV infection after 30 days post challenge.

Treatment	Booster Injection (Days) ^a^	Efficacy of Protection	
		Survival (%)	RPS ^c^
Prime-Vaccination	NA ^b^	81	55
Booster	30	95	76

^a^, after prime-vaccination; ^b^, not applicable; ^c^, relative percent survival.

## Data Availability

Not applicable.
